# Target Points in Trastuzumab Resistance

**DOI:** 10.1155/2012/761917

**Published:** 2012-02-26

**Authors:** Sahar Shojaei, Mossa Gardaneh, Abbas Rahimi Shamabadi

**Affiliations:** National Institute of Genetic Engineering and Biotechnology (NIGEB), Pajoohesh Boulevard, Tehran-Karaj HWY, Km 15, Tehran 1497716316, Iran

## Abstract

Epidermal growth factor (EGF) family of receptors is involved in cell growth and differentiation. The human EGF2 (HER2) lacks natural ligands, and correlation between HER2 levels and carcinogenesis makes the receptor an ideal candidate for targeted therapy in breast cancer. Trastuzumab is a humanized antibody applied against HER2-positive breast tumors in clinic. Metastatic tumors respond well to trastuzumab therapy for the first year, but development of antibody resistance helps the tumors to regrow allowing the disease to progress. Trastuzumab resistance is shaped via a range of intracellular signaling pathways that are interconnected and share in key effector molecules. Identification of a common node central to these resistance pathways could provide an ultimate solution for trastuzumab resistance in breast and other cancers.

## 1. Introduction

Breast cancer (BC) originates from the epithelial cells of the breast tissue that line the terminal duct lobular unit. BC is the most common cancer type that affects world population. More than 180,000 new cases of BC were diagnosed in 2008 in the United States alone [1]. Over 40,000 of these diagnosed cases resulted in death, mostly in women [1]. BC in women is the most commonly diagnosed cancer that accounts for 26% of all new cancer cases [2].

Well-known growth signaling pathways contribute to generation and progression of BC among other cancer types by promoting cell growth and proliferation [3]. These signaling pathways are promoted by a number of membrane-bound and intracellular receptors. The gene expression and biological activities of these receptors may have great impact on BC tumor initiation, progression, relapse, and prevention or treatment. Estrogen receptor (ER), progesterone receptor (PR), rearranged during transfection (RET), and human epidermal growth factor 2 (HER2) are the main membrane-bound receptors playing key roles in BC.

Hormone therapy is directed against ER that is expressed in 70% of BC tumors. Antibody therapy, on the other hand, was initiated with development of trastuzumab (TZMB) that specifically targets HER2 in 20 to 30% of BC cases where HER2 is strongly present. Resistance to hormone therapy and TZMB therapy are two major hurdles in current clinical BC therapy. In this paper, we will focus on the main causative sources of TZMB therapy and recent developments in exploration of key molecules that hold promise for eradication of this resistance.

## 2. HER2 Receptor

The HER/EGF family of receptors consists of four cell-surface receptors named HER1 (erbB1), HER2 (erbB2), HER3 (erbB3), and HER4 (erbB4) [4, 5]. These receptors are involved in cell growth, differentiation, and survival. They are activated by a ligand that causes heterodimerization of these receptors so that a cascade of phosphorylation and signal transduction events is initiated leading to transcription of specific genes involved in cell proliferation and survival [6]. Receptor dimers that contain HER2 produce stronger and more prolonged signal transduction event than those dimmers formed by other HER receptors [4–6].

The gene encoding HER2/neu (erbB2) is a protooncogene located in chromosome 17q21 and encodes a 185-kD transmembrane glycoprotein with tyrosine kinase activity [4, 5]. HER2 intracellular domain has a terminal carboxy segment autophosphorylation of which transmits the extracellular signal into an intracellular signal transduction event. In 20–30% of breast cancer tumors, the HER2 receptor is either amplified, overexpressed, or undergoes both events [4, 7]. Receptor overexpression is generally due to gene amplification, with one study reporting up to a 25-fold increase in HER2 copy number [8]. Tumors with HER2 overexpression generally have a poor disease-free survival [9, 10].

High levels of HER2 have strong correlations with the pathogenesis, and prognosis of breast cancer [11, 12]. Overexpression of the HER2 protein is detectable both in the primary tumors and in metastatic sites [13] indicating the effectiveness of anti-HER2 therapy in all disease locations.

HER2 is distinguished from other HER family members by lack of a natural ligand which makes the molecule a suitable therapeutic candidate. In addition, a strong correlation exists between HER2 levels and carcinogenesis [14, 15]. High levels of HER2 found in cancer cell membranes compared to those of normal cells and HER2 expression in both primary tumors and metastatic sites have made HER2 inhibitors important for breast cancer therapy [16, 17].

Research on stem cells as the initiators of breast cancer development has elucidated the status and function of the HER2 receptors in BC stem cells (BCSCs). Studies on patient samples show a significant correlation between HER2 overexpression and the expression of aldehyde dehydrogenase 1 (ALDH1) a key marker for BCSCs [18]. HER2 overexpression also acts as a driving force for breast stem cell malignancy, mammary tumorigenesis and invasion [19]. Epithelial cells isolated from normal breast form mammospheres that are nonadherent and spherical morphologies [20]. HER2 overexpression increases mammosphere formation by BCSCs [19]. Generation of mammospheres is an indication of increased self-renewal in these stem cells, whereas the size of these colonies that indicates proliferation of progenitors is also increased by HER2. The report by Korkaya and coworkers indicates that HER2 not only increases stem-cell number but also upregulates the expression of stem cell-related genes including Oct3/4, Nothc1, Notch2, Jag1, Gli1 in Aldefluor^+^ cells. HER2 also activates the Akt growth pathway in these stem cells [19].

## 3. HER2-ER Crosstalks

Crosstalks between BC cell receptors are important for full implementation of their biological activities. HER2 interacts with ER in a variety of approaches. Estradiol as an ER ligand induces a signal transduction cascade that trans-activates HER2 via SRC tyrosine kinase and the matrix metalloproteases MMP-2 and MMP-9 [21]. ER/HER2 interactions activate ERK and MAPK pathways and the two receptors equally contribute to ERK amplification in human mammary epithelial cells [22]. Therefore, ER and HER2 act in synergy to promote aberrant breast tumor growth. Co-expression of HER2 and ER co-activator A1B1 at high levels enhances tamoxifen resistance. In fact, in MCF7 tumor cell line overexpressing HER2 and A1B1, tamoxifen promoted tumorigenicity by inducing tumor cell growth both *in vitro* and *in vivo* [23].

## 4. HER2 Inhibition by Trastuzumab

HER2 was one of the first receptors targeted with the invention of its specific monoclonal antibody TZMB [24]. The encouraging preclinical results of this therapy took TZMB to clinic [25]. Currently, TZMB is used alone or as adjuvant with chemotherapy for treatment of breast cancer, especially in advanced stages of the disease.

TZMB is a recombinant humanized monoclonal antibody directed against the extracellular domain of HER2 inhibiting receptor dimerization [24, 26]. The mechanism of TZMB action on HER2-overexpressing tumors is not fully known, but experimental data exist that indicate the antibody influences several intracellular pathways all in favor of tumor cell death [27]. The multiple effects of TZMB can be explained by HER2 involvement in multiple signaling pathways. HER2 induces PI3K and MAPK and so activates downstream growth/proliferation pathways. TZMB-HER2 interaction inhibits these cascades leading to increased levels of p27^Kip^ ultimately arresting cell cycle and inducing apoptosis [28]. Induction of antibody-dependent cellular cytotoxicity (ADCC) leading to cancer cell lysis [24, 29] and inhibition of angiogenesis are two other effects of TZMB in favor of BC treatment. The overall response rate to TZMB monotherapies is ~26% compared to 40–60% when a TZMB-chemotherapy combination is applied [25, 30, 31].

An important function of TZMB on HER2 is to prevent receptor degradation and cleavage. This function can be shown by examining serum samples in TZMB-treated patients who have reduced levels of HER2 shedding in their serum [32]. TZMB-mediated reduction of HER2 levels in patients' serum has been accompanied with improved progression-free survival and so considered an indicative of host response to the antibody therapy [33].

In addition to its effect on HER2 dimerization and cleavage, TZMB can cause HER2 internalization and degradation as shown in HER2-amplifying BC cell lines and tumor samples treated with the antibody [34, 35]. It is not clear, however, if TZMB actually contributes to HER2 gene downregulation, since some studies report unchanged levels of HER2 upon TZMB treatment [29, 36].

## 5. Mechanisms of Trastuzumab Resistance

Resistance to TZMB therapy occurs in both *de novo* (intrinsic) and acquired forms. The *de novo* resistance results from genetic changes in receptor tyrosine kinases (RTKs) and their downstream cellular pathways. The main genetic changes in this case include deficient PTEN [37] or mutated *PIK3CA* genes [38] that cause the constitutive activation of the PI3K pathway, and expression of a truncated, rather than full length, HER2 receptors named p95HER2 that lack extracellular domain needed for TZMB binding [39].

The acquired form of TZMB resistance is caused mainly by shaping compensatory kinase signaling pathways alternative to HER2-mediated pathways leading to BC cell growth and TZMB insensitivity. There are also genetic alterations that are source of both *de novo* and acquired forms of resistance. Below, we outline some of the main mechanisms behind TZMB resistance that we have summarized in Table 1.

### 5.1. Truncated HER2 Versions

In addition to genetically shortened HER2 receptors, cleavage by metalloproteases generates a truncated HER2 receptor lacking the extracellular domain required for TZMB binding [57]. The truncated receptor will retain its kinase activity undisturbed in the absence of any blocker [39]. The truncated HER2 versions might be a source of *de novo* TZMB resistance because HER2 cleavage can be blocked by TZMB [32] that prevents resistance to be acquired in the course of treatment.

### 5.2. HER2 Masking

TZMB can be deprived of reaching and effectively binding HER2 due to function of the membrane-bound glycoprotein mucin-4 (MUC4). MUC4 contributes to BC progression by protecting cancer cells from immune recognition, inducing tumorigenicity and metastasis, suppressing apoptosis, and activating HER2 [43]. Using its ASGP-2 subunit that contains an EGF-like domain, MUC4 can directly bind HER2 and induce HER2 phosphorylation. Through this interaction, MUC4 competes with TZMB for HER2 binding as shown in BC cell lines overexpressing MUC4 [44]. Studies on patient-derived cell lines with HER2 amplification and *de novo* resistance to TZMB demonstrated an inverse correlation between HER2-binding capacities of MUC4 and TZMB [45]. These observations suggested that HER2 epitopes are masked by MUC4 which causes steric hindrance of trastuzumab-HER2 interaction and shapes antibody resistance.

### 5.3. Signaling by Other HER Family Members

The four members of EGFR type 1 growth factor RTK family, namely, EGFR, HER2, HER3, and HER4 tend to dimerize with HER2 as a partner of their choice when induced by their relevant ligands [46]. This heterodimerization results in RTK activity that is echoed by activation of downstream signaling, components via the MAPK and PI3K pathways. The function of TZMB is to prevent HER2-mediated signaling but it cannot effectively inhibit signaling promoted by other HER receptors. Therefore, heterodimers or homodimers constituted by EGFR and HER3 may induce PI3K/MAPK pathways. Growth inhibition induced by TZMB can also be prevented by the activity of endogenous HER family ligands that induce HER2/HER3 and HER2/EGFR signaling [47, 48]. These observations indicate that the overall efficacy of TZMB can be dictated by the endogenous status of EGFR family members, their ligands, and inhibitors. Determination of the levels of these molecules within BC cells could help to improve TZMB function. Another solution is to block crosstalks between RTKs using bispecific antibodies or combination of TZMB and lapatinib or TZMB and pertuzumab that seem to confer more improvement to recipient patients [49, 50].

### 5.4. IGF-1R Signaling

The insulin-like growth factor-1 receptor (IGF-1R) is a transmembrane tyrosine kinase receptor predominantly expressed in human BCs and involved in proliferation and metastatic dissemination [58]. IGF-1R interacts with HER2 and contributes to TZMB resistance in BC cells [59]. Since both IGF-1R and HER2 promote common downstream pathways of cell growth and proliferation, the study shows that their cooverexpression inhibits TZMB-mediated growth inhibition [60]. Therefore, it is likely that tumors coexpressing the two receptors resist TZMB-mediated therapy. On the other hand, drugs acting as IGF-1R antagonists such as IGFBP3 that blocks IGF resensitize resistant BC cells to the antibody [61]. Like other receptor kinases, IGF-1R is also dependent on PI3K/Akt pathway for its biological functions including p27^Kip1^ degradation [28]. IGF-1R has been further found to induce phosphorylation of HER2, an activity of IGF-1R observed only in TZMB-resistant cells [62]. Restoration of TZMB sensitivity in BC cells upon inhibition of IGF-1R signaling either by antibody-mediated blockage or IGF-1R tyrosine kinase inhibition introduces signaling pathways downstream of IGF-1R as therapeutic targets to break antibody resistance.

### 5.5. PTEN

Mutated or downregulated PTEN leads to its loss of function, a phenomenon described in nearly 50% of breast cancers [63]. Since PTEN has inhibitory effects on PI3K, loss of PTEN function constitutively maintains the activity of the PI3K/Akt pathway [37] that inhibits cell-cycle arrest and apoptosis mediated by TZMB [40]. Patients with BC tumors that lack PTEN expression but overexpress HER2 more poorly respond to TZMB therapy than those patients with normal PTEN expressed by their tumor cells [37]. Therefore, PTEN loss could provide another indication for TZMB resistance. In fact, in those patients with tumors reluctant to respond to TZMB therapy but with high-level PITEN expression, inhibition of PI3K/Akt pathway could provide another therapeutic opportunity in clinic, as shown by TZMB and lapatinib response upon PTEN loss and PI3K activation [64].

### 5.6. p27

The cyclin-dependent kinase-inhibiting protein p27^Kip1^ mediates growth inhibitory effects of trastuzumab [42]. Trastuzumab has positive effects on p27^Kip1^ half-life [65] and supports formation of p27^Kip1^-cyclin-dependent kinase 2 (CDK2) complexes that arrest cell cycle at G1 [66]. In fact, inhibition of p27^Kip1^ expression blocks trastuzumab-mediated growth arrest in HER2-overexpressing BC cells [40]. Overexpression of p27^Kip1^ or its induction by the proteasome inhibitor MG132 restored trastuzumab sensitivity that highlights the critical role of p27^Kip1^ in trastuzumab function [28]. On the other hand, CDK2 inhibitors result in reduced cell proliferation and enhanced apoptosis *in vitro* and reduced tumor growth in xenografts resistant to trastuzumab [67].

### 5.7. PI3K/Akt Pathways

Earlier we outlined the importance of PTEN loss in constitutive activation of the PI3K/Akt pathway leading to TZMB resistance. The pathway can also be activated via PI3K mutations. PIK3CA that encodes the catalytic subunit of PI3K is a gene frequently mutated in breast cancer and promotes TZMB insensitivity in BC cells *in vitro* [41]. *In vivo* studies also point to the anti-TZMB role played by mutated versions of PIK3CA when applied with reduced PTEN expression [38]. In parallel, HER2-amplified breast cancer cell lines containing PIK3CA hotspot mutations more significantly resisted TZMB than mutant-free cells [68]. Therefore, the PI3K/Akt pathway provides another landmark for TZMB efficacy.

### 5.8. Cyclin E Amplification/Overexpression

The overexpression of the cyclin E in BC cell lines and tumor samples has been proposed as a marker of poor clinical outcome [69]. There are conflicting reports on relationships between cyclin E and HER2. A previous study indicated reduction of cyclin E levels upon HER2 downregulation and HER2 inhibition and so suggested cyclin E regulation by HER2 [70]. It is now emerging from a recent report that cyclin E might have an impact on HER2 function in BC cells [67]. In this paper, genome-wide analysis of TZMB-resistant BC cell lines and tumor cells indicates that cyclin E is amplified and so overexpressed in these cell samples. In this study, the amplified cycline E gene was found to worsen clinical outcome and reduce progression-free survival. While cycline E overexpression enhanced TZMB resistance both *in vitro* and *in vivo*, suppression of cyclin E activity highly reduced cell proliferation and induced apoptosis among cyclin E-amplifying BC cells. Cyclin E suppression mediated by CDK2 inhibition reduced tumor growth among xenografts that were insensitive to TZMB [67]. The use of CDK inhibitors could be a novel approach to counteract antibody resistance in targeting HER2.

### 5.9. Alternative Signaling Pathways

**Figure 1:**
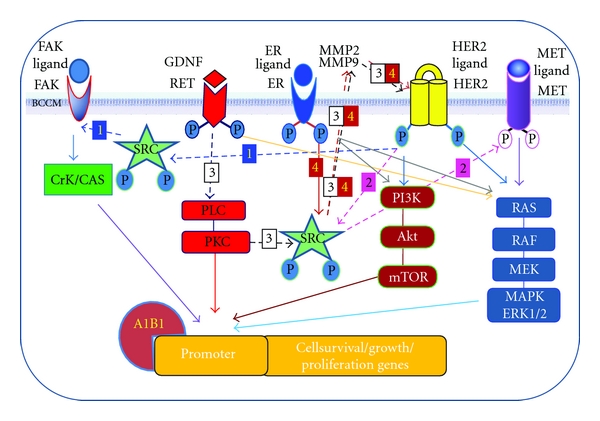
Growth pathways in breast cancer. Key receptors are shown phosphorylated upon ligand binding, a chemical change that triggers downstream growth and proliferation pathways. Receptor crosstalks between receptors are shown by dotted arrows that can be chased by numbers: 1: Her2-FAK, 2: Her2-Met, 3: ER-Her2, 4: Ret-Her2. BCCM: breast cancer cell membrane; MMP: matrix metalloproeinases; A1B1: ER coactivator. For details of signaling pathways see the text.

HER2 crosstalks with ER and with RTKs, as we discussed in Section 4, are critical for activation of signaling pathways leading to BC tumor growth. Ret tyrosine kinase interacts with SRC kinase upon activating protein kinase C (PKC) and induces upregulation of many growth pathways through the function of SRC as key inducer of TZMB resistance discussed below. More importantly, SRC mediates Ret/Her2 crosstalks: phosphorylated in its SH2 domain via GDNF/Ret downstream PLC/PKC pathway, SRC induces Her2 phosphorylation by matrix metalloproeinases MMP2 and MMP9 (Figure 1) [51].

Ret is also in crosstalk with ER for ER gene overexpression [54] and receptor stabilization [55]. In BC cell lines resistant to tamoxifen treatment, HER2 signaling pathways are selected against Ret/ER pathways to promote cell growth whereas targeting Ret restores drug sensitivity [56]. Phosphorylated SRC activates alternative receptors RTKs MET and FAK both of which promote mitogenesis and cancer cell growth (Figure 1) [52, 53]. MET downstream pathway includes RAS, RAF, and MAPK/ErK1/2, whereas FAk induces Crk/CAS and deregulation of both pathways can promote cancer cell growth alternative to other receptors.

The capacity of BC cells in switching from one receptor to another for growth promotion suggests that Ret, like other RTKs, can induce alternative growth signaling in support of resistance to TZMB therapy. Although the function of Ret in TZMB-resistant cells and tumor samples has not been reported, Ret inhibition might be a novel strategy to overcome such resistance.

### 5.10. Immune Response Induction

TZMB-mediated growth arrest and BC cell death are partially due to the induction of immune responses by the antibody. Antibody-dependent cellular cytotoxicity (ADCC) is induced by TZMB and other antibodies targeting HER2 leading to apoptosis in several BC cell lines [29, 45, 71].

Anti-HER2 antibody-mediated BC cell death occurs via natural killer (NK) cells that, by expressing the Fc gamma receptor, interact with the Fc domain of the antibody [72]. The importance of this interaction has been shown in a xenograft model of mice lacking Fc receptor where TZMB could only partially inhibit tumor growth [72]. These findings indicated the role of the immune system in sustaining anti-HER2 therapies.

### 5.11. SRC: A Proposed Central Node

Based on reports that SRC is commonly activated in EGFR/IGF-1R-overexpressing and PTEN-deficient cells, the molecule appears to play a central role in development of resistance against HER2-mediated antibody therapy. This is particularly important in terms of SRC involvement in common events downstream of key growth signaling pathways and so SRC function as a common node in promoting TZMB resistance.

SRC encodes a nonreceptor tyrosine kinase that in its active form contributes to many hallmarks of cancer including cell proliferation, migration, and angiogenesis [73, 74]. The SRC family contains four SRC homology domains called SH and a C-terminal segment that has a negative regulatory tyrosine residue (Tyr530) [73]. SRC is activated upon phosphorylation that is catalyzed by protein tyrosine phosphatase alpha and SH-containing phosphatases SHP1/SHP2 [73]. On the other hand, SRC kinases including RTKs EGFR, HER2, FGFR, PDGFR, and VEGFR activate the protein by phosphorylating it [75].

SRC is hyperactivated in TZMB BC cell models and has implications for TZMB resistance. For instance, tumor necrosis factor **α** (TNF**α**) induces HER2 phosphorylation in breast cancer cells via c-Src activation [76]. Another example is erythropoietin receptor (EpoR) that induces MAPK/Erk and PI3K/Akt pathways [77]. BC cell lines coexpressing EpoR and HER2 induce TZMB resistance upon treatment with recombinant erythropoietin that interacts with phosphorylated EpoR [78]. Activated SRC and inactivated PTEN were found to be behind increased TZMB resistance [78].

SRC activation occurs mainly due to phosphorylation of Tyr419 and Tyr416 residues [75]. For example, overexpression of RTKs EGFR and IGF-IR induces Tyr416 phosphorylation and promotes antibody resistance whereas siRNA-mediated suppression of these kinases reduces resistance [79]. SRC has been shown to inhibit PTEN activity by inducing tyrosine phosphorylation and blocking PTEN membrane localization [37, 80] suggesting that SRC and PTEN may regulate each other to promote TZMB resistance. Based on these reports, Zhang et al. further examined the role of SRC in *de novo* insensitivity of BC tumor cells to trastuzumab treatment by inhibiting PTEN.

Antisense- or shRNA-mediated downregulation of PTEN induced SRC Tyr416 phosphorylation, SRC activation, and ultimately elevated TZMB resistance, whereas induction of PTEN phosphatase activity directly dephosphorylated SRC Tyr416 residue and so abolished SRC activity [79]. These observations indicate that the loss of PTEN phosphatase activity induces SRC activation and so implicates SRC in shaping *de novo *TZMB resistance in PTEN-deficient cells [81]. Indeed, hyperphosphorylation of Tyr416 that increases SRC activity is an inducing factor for TZMB resistance, whereas Tyr416 dephosphorylation sensitizes BC cells to TZMB-mediated growth arrest.

The activity of SRC *per se* and its role in antibody resistance was examined using both SRC small-molecule inhibitor saracatinib and SRC shRNA molecules. Inhibition of SRC halted EGF-induced EGFR dimerization and inhibited phosphorylation of an EGFR residue known to be SRC-dependent phosphorylation site [81] and an autophosphorylation site within EGFR [79]. EGFR phosphorylation was also inhibited in TZMB-resistant cells treated with saracatinib. Moreover, treatment with TZMB further activated SRC due to the transient induction of HER2 dimerization/phosphorylation by TZMB in resistant cells. In contrast, inactivation of SRC by saracatinib or SRC shRNAs diminished TZMB-induced transient phosphorylation of HER2 as well as HER3 [79]. These observations indicate that SRC hyperactivity in TZMB-resistant BC cells promotes a positive feedback cycle where SRC activates EGFR, HER2, and HER3 which, in turn, can activate SRC. Overall, the report by Zhang et al. highlights the increasing central role of SRC as common converging point for all the intracellular forces that contribute to shaping and re-gaining BC cell resistance toward HER2-directed treatments specially TZMB therapy.

## 6. Conclusions and Future Therapies

TZMB is an antibody of choice for HER2-directed BC therapies. In patients with metastatic, HER2-positive breast cancer, TZMB administered as an adjuvant combined with chemotherapy shows significant clinical benefits [25, 80]. However, the majority of HER2-positive patients do not respond to TZMB due to* de novo *or acquired resistance. This review outlined main molecules and major pathways that causatively contribute to TZMB resistance. Abnormalities in HER2 structure/function and in downstream signaling pathways as well as RTK crosstalks have been suspected causes as evidenced by examination of BC cells and tumor samples *in vitro* and *in vivo*. The most recent studies pinpoint to SRC acting as a downstream signaling effector and a central common node in mediating TZMB resistance.

Receptor crosstalks blocked by bispecific antibodies or by TZMB combined with lapatinib or pertuzumab have shown better clinical outcomes compared to single antibody treatment. Therefore, a multipronged strategy capable of effectively blocking the HER2 signaling network is needed to inhibit HER2 homodimerization and HER2-RTK heterodimerization, so HER2-dependent malignant BC tumors become fully controllable. Compensation for loss of expression or activity of several molecules involved in TZMB resistance may partially reverse resistance and resensitize BC cells and tumors to the antibody. However, disarming common nodes, for example, by inhibition of SRC expression and or function holds promise for universally combating resistance and controlling HER2-positive BC tumors. Coinhibition of SRC and RTKs that promote bypassing growth pathways such as Ret, FAK, and Met could provide a global coverage against TZMB resistance.

## Figures and Tables

**Table 1:** Mechanisms of *de novo* and acquired trastuzumab resistance. BCSC: breast cancer stem cell; TZMB: trastuzumab; Ab: antibody; mAb: monoclonal antibody; RTK: receptor tyrosine kinase. See text for more details.

Type of resistance	Molecule	Role in cell growth		Role in TZMB resistance	Therapeutic intervention	References
*De novo* or genetical resistance	PTEN	Inhibition of PI3K pathways and control of cell growth		Mutated PTEN unresponsive to TZMB, constitutive activation of PI3K pathway	siRNA inhibition of Akt	[37, 40]
	PIK3CA	Encoding PTEN-resistant catalytical subunit of PI3K		PI3 unresponsive to PTEN, constitutive activation of PI3K pathway	siRNA inhibition of Akt	[38, 41]
	P95HER2	Truncated HER2 with kinase activity		HER2 cleavage blocked by TZMB	RTKs inhibitor: lapatinib	[32, 39]
	P27	CDK2 inhibition and cell cycle arrest in G1		Induction of cell growth upon p27 inhibition or mutation	p27 overexpression, proteasome inhibition, CDK2 inhibition	[40, 42]

Acquired resistance (shaped by compensatory kinase signaling pathways)	MUC4	Inhibition of immune recognition, HER2 activation		HER2 masking from TZMB, induction of HER2 phosphorylation	RTKs inhibitor: lapatinib	[43–45]
	HER family members (RTKs)	Heterodimerization with Her2 and activation of growth signaling pathways		RTKs activation of alternative signaling pathways bypassing HER2	Bispecific Abs or TZMB combined with other mAbs against RTKs	[46–50]
	Alternative signaling pathways	RET	GDNF-mediated activation of PLC cell growth pathway	Crosstalk with HER2 via SRC activation, cross talk with Met	Concomitant use of TZMB and siRNA against RET and/or SRC	[51]
		FAK	BCSC growth by activation of Crk/CAS growth pathway	Crosstalk with HER2 via SRC activation	Combination of TZMB and SiRNA against SRC	[52]
		Met	Cell mitogenesis and morphogenesis by activation of MAPK growth pathway	Crosstalk with HER2 via SRC activation	Combination of TZMB and SiRNA against SRC	[53]
		ER	Estradiol-mediated growth by activation of PLC pathway	Crosstalk with RET and with HER2 via Src activation	Concomitant use of TZMB, Tamoxifen, and SRC-SiRNA	[54–56]
